# Evaluation of Transduction Properties of an Adenovirus Vector in Neonatal Mice

**DOI:** 10.1155/2015/685374

**Published:** 2015-05-13

**Authors:** Shunsuke Iizuka, Fuminori Sakurai, Kahori Shimizu, Kazuo Ohashi, Shin-ichiro Nakamura, Masashi Tachibana, Hiroyuki Mizuguchi

**Affiliations:** ^1^Laboratory of Biochemistry and Molecular Biology, Graduate School of Pharmaceutical Sciences, Osaka University, 1-6 Yamadaoka, Suita, Osaka 565-0871, Japan; ^2^Laboratory of Regulatory Sciences for Oligonucleotide Therapeutics, Clinical Drug Development Unit, Graduate School of Pharmaceutical Sciences, Osaka University, 1-6 Yamadaoka, Suita, Osaka 565-0871, Japan; ^3^Faculty of Pharmacy, Osaka Ohtani University, 3-11-1 Nishikiorikita, Tondabayashi, Osaka 584-8540, Japan; ^4^Laboratory of Drug Development and Science, Graduate School of Pharmaceutical Sciences, Osaka University, 1-6 Yamadaoka, Suita, Osaka 565-0871, Japan; ^5^Laboratory of Hepatocyte Differentiation, National Institute of Biomedical Innovation, 7-6-8 Saito asagi, Ibaraki, Osaka 567-0085, Japan; ^6^Research Center of Animal Life Science, Shiga University of Medical Science, Setatsukinowa, Otsu, Shiga 520-2121, Japan; ^7^iPS Cell-Based Research Project on Hepatic Toxicity and Metabolism, Graduate School of Pharmaceutical Sciences, Osaka University, 1-6 Yamadaoka, Suita, Osaka 565-0871, Japan; ^8^The Center for Advanced Medical Engineering and Informatics, Osaka University, 2-2 Yamadaoka, Suita, Osaka 565-0871, Japan

## Abstract

In gene therapy for congenital disorders, treatments during neonate and infant stages are promising. Replication-incompetent adenovirus (Ad) vectors have been used in gene therapy studies of genetic disorders; however, the transduction properties of Ad vectors in neonates and infants have not been fully examined. Accordingly, this study examined the properties of Ad vector-mediated transduction in neonatal mice. A first-generation Ad vector containing a cytomegalovirus (CMV) promoter-driven luciferase expression cassette was administered to neonatal mice on the second day of life *via* retro-orbital sinus. The highest Ad vector genome copy numbers and transgene expression were found in the neonatal liver. The neonatal heart exhibited the second highest levels of transgene expression among the organs examined. There was an approximately 1500-fold difference in the transgene expression levels between the adult liver and heart, while the neonatal liver exhibited only an approximately 30-fold higher level of transgene expression than the neonatal heart. A liver-specific promoter for firefly luciferase expression conferred a more than 100-fold higher luciferase expression in the liver relative to the other organs. No apparent hepatotoxicity was observed in neonatal mice following Ad vector administration. These findings should provide valuable information for gene therapy using Ad vectors in neonates and infants.

## 1. Introduction

The mechanisms of several congenital diseases have been gradually revealed; however, efficient treatment protocols have not been established for most congenital diseases, including X-linked severe combined immune deficiency (X-SCID), hemophilia, and lysosomal storage disorder. The existing supportive treatments, such as enzyme replacement treatment, often do not achieve sufficient therapeutic effects. Moreover, several congenital diseases are characterized by life-threatening symptoms in the early stage of life [1, 2]. Radical treatment during neonate or infant stage is required for these patients, and gene therapy is a promising candidate for such treatment.

There are several advantages to gene therapy in neonates and infants. (i) Neonates and infants have immature immune systems, making it possible to circumvent the immune reactions against gene delivery vectors and transgene products that reduce therapeutic efficiency in adults. (ii) Neonates and infants have lower body weight than adults, and thus efficient therapeutic effects can be achieved at lower vector doses. (iii) Neonates and infants have higher stem/progenitor cell ratios, and thus transduction into stem/progenitor cells may occur more efficiently than in adults. Numerous gene therapy trials have been conducted using animal model of neonatal disease and viral vectors, such as adeno-associated virus [3], retrovirus [4], and a lentivirus vectors [5].

Adenovirus (Ad) vectors are among the most commonly used viral vectors in gene therapy clinical trials because they have the following advantages. (i) They can achieve a 1000-fold higher-titer vector stock compared with other viral vectors, such as adeno-associated virus, retrovirus, and lentivirus vectors, at an equivalent scale. (ii) They have a large packaging capacity (about 8.0 kb). (iii) They do not exhibit genetic toxicity because they do not integrate the transgene into the host genome. (iv) They can deliver transgene into the nuclei of not only dividing cells but also nondividing cells with the highest efficiency among the gene delivery vectors. On the other hand, Ad vectors also have a number of disadvantages, including their low transduction efficiency in cells lacking coxsackievirus-adenovirus receptor (CAR) and their induction of innate immune responses. In order to overcome these disadvantages of the first-generation Ad vectors, next-generation Ad vectors, including fiber-mutant Ad vectors and helper-dependent Ad vectors, have been developed [6–8].

Ad vectors have been used in gene therapy clinical trials of adult patients with various diseases, including several types of cancers and cardiac diseases, and promising results have been reported [9, 10]. These various advantageous properties of Ad vectors led us to consider that they might also be suitable for gene therapy in neonates and infants; however, the transduction properties of Ad vectors in neonates and infants have not been fully examined.

In this study, in order to evaluate the properties of Ad vector-mediated transduction in neonates, a first-generation Ad vector was systemically administered to neonatal mice on the second day of life. Then, the biodistribution of the Ad vector, the Ad vector-induced toxicity, and the Ad vector-induced immune responses were examined. The results should be highly informative for gene therapy in neonates and infants as well as basic studies using neonatal mice.

## 2. Materials and Methods

### 2.1. Cell Culture

HEK293 cells (a human embryonic kidney cell line) were cultured in Dulbecco's modified Eagle's medium (Wako Pure Chemical, Osaka, Japan) supplemented with 10% fetal calf serum (FCS), 2 mM glutamine, and antibiotics.

### 2.2. Plasmids and Replication-Incompetent Ad Vectors

**Figure 1:**
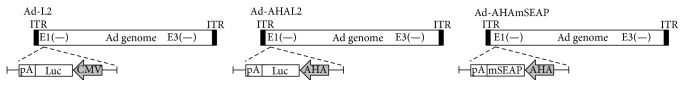
Schematic diagrams of the replication-incompetent Ad vectors used in this study. A luciferase or murine secreted embryonic alkaline phosphatase (mSEAP) expression cassette was inserted into the E1-deleted region in the Ad vector genome. AHA, a synthetic promoter composed of an apolipoprotein E enhancer, the hepatocyte control region, and a human alpha1-antitrypsin promoter; CMV, cytomegalovirus promoter; ITR, inverted terminal repeat.

A first-generation Ad vector containing a cytomegalovirus (CMV) promoter-driven firefly luciferase expression cassette (Ad-L2) [11] and an Ad vector containing a synthetic liver-specific promoter composed of an apolipoprotein E enhancer, the hepatocyte control region, and a human alpha1-antitrypsin promoter- (AHA promoter-) [12] driven murine secreted embryonic alkaline phosphatase (mSEAP) expression cassette (Ad-AHAmSEAP) [13] was previously constructed. An Ad vector containing an AHA promoter-driven firefly luciferase expression cassette was prepared by an improved* in vitro* ligation method [14, 15]. An AHA promoter-driven firefly luciferase-expressing plasmid, pAHA-L2, was constructed using pHMRSV6 [15], pBS-ApoEHCR-hAATp-hFIX-Int-bpA [12], and pCMVL1 [11]. pAHA-L2 was digested with I-*Ceu*I/PI-*Sce*I, and subsequently ligated with I-*Ceu*I/PI-*Sce*I-pAdHM4 [14], resulting in pAdHM4-AHAL2. pAdHM4-AHAL2 was digested with* Pac*I to release the recombinant viral genome and was transfected into 293 cells plated on 60 mm dishes. Ad vectors were propagated in 293 cells, purified by two rounds of cesium chloride-gradient ultracentrifugation, dialyzed, and stored at −80°C. The virus particles (VPs) were determined using a spectrophotometric method [16]. Biological titers were measured using an Adeno-X-rapid titer kit (Clontech, Mountain View, CA). The ratio of the particle-to-biological titer was between 6.5 (minimum) and 15.6 (maximum) for each Ad vector used in this study. The Ad vectors used in this study are illustrated in Figure 1 and listed in Table 1.

### 2.3. Mice and Animal Procedures

C57BL/6 mice aged 5–7 weeks were obtained from Nippon SLC (Hamamatsu, Japan). Second day of life (DOL 2) mice from a mating of C57/BL6 mice were used as the neonatal animals. Female mice aged 5–7 weeks were used as the adult animals. Ad vectors were injected at a dose of 5.9 × 10^11^ infectious units (IFU)/kg* via* retro-orbital sinus to both neonatal and adult mice, if not otherwise specified. Ad vector injection was performed in a total volume of 50 *μ*L in neonatal mice and 200 *μ*L in adult mice. Blood samples were collected by retro-orbital bleeding in adult mice or venous bleeding in neonatal mice. All animal experimental procedures used in this study were performed in accordance with the institutional guidelines for animal experiments at Osaka University.

### 2.4. Analysis of Luciferase Expression and Biodistribution of Ad Vectors in the Organs

Ad-L2 and Ad-AHAL2 were administered to neonatal and adult mice at doses of 5.9 × 10^11^ IFU/kg and 1.2 × 10^11^ IFU/kg* via* retro-orbital sinus. These titers are approximately equal to 1 × 10^10^ IFU/mouse and 0.2 × 10^10^ IFU/mouse for 5-week-old mice, respectively, which have a body weight of approximately 17 g. Two days after administration, the organs were recovered and homogenated as previously described [17]. Ad vector accumulation in the organs was evaluated by real-time PCR analysis 2 days after administration as previously described [18].

### 2.5. *In Vivo* Imaging of Ad Vector-Mediated Luciferase Expression

Neonatal mice were administered Ad-AHAL2 at a dose of 5.9 × 10^11^ IFU/kg. Mice were intraperitoneally administered D-luciferin potassium salt (Wako Pure Chemical), as a luciferase substrate, at a dose of 150 mg/kg at the indicated time points. Five minutes later, the isoflurane-anaesthetized mice were imaged using NightOwl (Belthold, Bad Wildbad, Germany). The visual output represents the counts as a pseudocolor image where the maximum is red and the minimum is blue. At each time point, a region of interest was generated surrounding each animal (excluding the tail) to quantify the total counts by luciferase activity.

### 2.6. Evaluation of Ad Vector-Mediated* In Vivo* Toxicity following Systemic Administration

Neonatal mice were administered Ad-AHAL2 at a dose of 5.9 × 10^11^ IFU/kg. Blood samples were collected on the indicated day points and placed on ice for 1 h. The serum was collected following centrifugation at 7000 g at 4°C for 15 min. The serum alanine aminotransferase (ALT) and aspartateaminotransferase (AST) levels in the serum were determined using a transaminase-CII kit (Wako Pure Chemical). For the histopathological examination of liver sections, the livers were recovered from mice 2 days after Ad vector administration. The livers were washed, fixed in 10% buffered formalin (Wako Pure Chemical), embedded in paraffin, and processed for histology. For evaluation of Ad vector-mediated cardiac toxicity, the serum creatine kinase (CK) levels were determined using a Creatine Kinase Assay kit (BioAssay Systems, Hayward, CA).

### 2.7. Cytokine mRNA Levels in the Livers and Spleens after Ad Vector Administration

Neonatal and adult mice were administered Ad-AHAL2 at a dose of 5.9 × 10^11^ IFU/kg. Three hours after administration, total RNA was extracted from the liver and spleen using ISOGEN (Wako Pure Chemical). The mRNA levels of the cytokines, including interleukin- (IL-) 6, IL-12, interferon- (IFN-) *γ*, and glyceroaldehyde-3-phosphatedehydrogenase (GAPDH), were determined by real-time RT-PCR using THUNDERBIRD SYBR qPCR Mix (Toyobo, Osaka, Japan). The protocol for thermal cycling consisted of 60 seconds at 95°C, followed by 40 cycles of 15 seconds at 95°C and 60 seconds at 60°C. The sequences of the primers were as follows: IL-6 forward, 5′-CTG CAA GAG ACT TCC ATC CAG-3′; IL6-Reverse, 5′-AGT GGT ATA GAC AGG TCT GTT G-3′; IL-12*β* forward, 5′-CTC AGA AGC TAA CCA TCT CCT G-3′; IL-12*β* reverse, 5′-CAC AGG TGA GGT TCA CTG TTT C-3′; IFN-*γ* forward, 5′-ATG AAC GCT ACA CAC TGC ATC-3′; IFN-*γ* reverse, 5′-TCT AGG CTT TCA ATG ACT GTG C-3′; GAPDH forward, 5′-CAA TGT GTC CGT CGT GGA TCT-3′; GAPDH reverse, 5′-GTC CTC AGT GTA GCC CAA GAT G-3′.

### 2.8. Anti-Ad Antibody Titers in the Serum following Intravenous Administration of an Ad Vector

Mice of various ages were intravenously administered Ad-AHAL2 at a dose of 5.9 × 10^11^ IFU/kg. The total injection volumes were 50 *μ*L for DOL 2 and DOL 7 mice, 100 *μ*L for DOL 14 and DOL 21 mice, and 200 *μ*L for 5- and 7-week-old mice. Blood samples were collected* via *retro-orbital bleeding or venous bleeding on the indicated day points. Serum samples were prepared as described above. Anti-Ad antibody titers in the serum were determined by enzyme-linked immunosorbent assay (ELISA) as previously described [13] with slight modifications. Serum samples were diluted to 1 : 640.

### 2.9. Transduction Efficiencies of an Ad Vector in the Preimmunized Mice

Neonatal and adult mice were intravenously administered Ad-AHAL2 at a dose of 5.9 × 10^11^ IFU/kg* via* retro-orbital sinus. Ad-AHAmSEAP was administered* via *the tail vein 21 days after the first injection. Two days after Ad-AHAmSEAP injection, blood samples were collected* via *retro-orbital bleeding and serum samples were prepared as described above. The mSEAP expression levels were determined using a Great EscAPe SEAP Chemiluminescence Kit, version 2.0 (Clontech).

### 2.10. Statistical Analysis

Statistical significance (*P* < 0.05) was determined using Student's *t*-test. Data are presented as means ± S.D.

## 3. Results

### 3.1. Ad Vector-Mediated Transduction in Neonatal Mice

**Figure 2:**
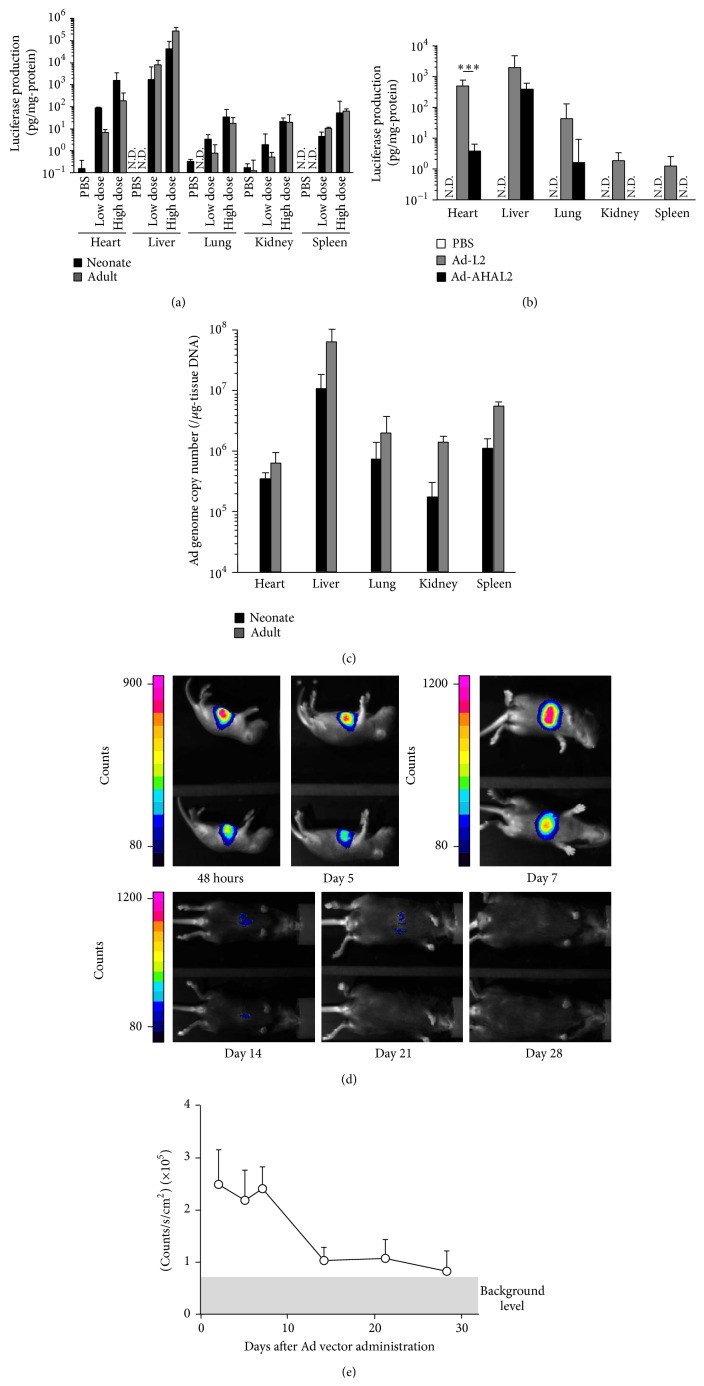
Transduction property of an Ad vector in neonatal mice. (a) Luciferase expression levels in the organs following systemic administration of Ad-L2. Neonatal and adult mice were administered Ad-L2 at a dose of 1.2 × 10^11^ IFU/kg (low dose) or 5.9 × 10^11^ IFU/kg (high dose)* via* retro-orbital sinus. Luciferase production was examined in each organ 48 h after Ad vector administration. (b) Liver-specific luciferase production in neonatal mice following systemic administration of Ad-AHAL2. Neonatal mice received Ad vectors at a dose of 5.9 × 10^11^ IFU/kg. Luciferase activities in the organs were measured as described in (a). (c) Ad vector genome copy numbers in the organs following systemic administration in the neonatal and adult mice. Mice were administered Ad-L2 at a dose of 5.9 × 10^11^ IFU/kg* via* retro-orbital sinus. Ad genome copy numbers in each organ were examined 48 h after injection. The PBS-administered group had <10^4^ Ad genome copies/*μ*g tissue DNA. (d, e) Duration of transgene expression following systemic administration of Ad-AHAL2 in neonatal mice.* In vivo* bioluminescence images are shown in (d). Bioluminescence intensity levels of the mice are graphically illustrated in (e). Neonatal mice were administered Ad-AHAL2 at a dose of 5.9 × 10^11^ IFU/kg* via* retro-orbital sinus. Pseudocolor scales were represented as counts unit. Note that the legend of the pseudocolor scale on day 5 is different from that on the other days (N.D., not detected) (*n* = 5-6 animals per group for all experiments).

In order to examine Ad vector-mediated transgene expression profiles in neonatal mice, Ad-L2, which is a first-generation Ad vector carrying a CMV promoter-driven firefly luciferase expression cassette, was administered to neonatal and adult mice at a doses of 1.2 × 10^11^ IFU/kg (low dose) and 5.9 × 10^11^ IFU/kg (high dose). As is the case with adult mice, Ad-L2 mediated a higher transgene expression in the liver than in the other organs examined in neonatal mice, although transgene expression levels in the neonatal liver were approximately 5-fold lower than those in the adult liver (Figure 2(a)). The levels of luciferase expression in the lung, kidney, and spleen were comparable between neonatal and adult mice. On the other hand, the neonatal heart exhibited about 10-fold higher luciferase expression, compared with the adult heart, following Ad-L2 injection. While the luciferase expression levels in the adult liver were approximately 1500-fold higher than those in the adult heart, only 30-fold higher luciferase expression was observed in the neonatal liver than in the neonatal heart. Pretreatment of Ad-L2 with coagulation factor X (FX), which plays a crucial role in hepatotropism of an Ad vector [19], before injection did not improve the transduction efficiencies of Ad-L2 in the liver of neonatal mice (Supplementary Figure 1 in Supplementary Material available online at http://dx.doi.org/10.1155/2015/685374). A high dose of Ad-L2 (5.9 × 10^11^ IFU/kg) mediated a luciferase expression pattern similar to that mediated by a low dose of Ad-L2 in the organs of neonatal and adult mice. We did not find significant differences in the transduction profiles of Ad-L2 in adult mice following tail vein injection and retro-orbital injection (Supplementary Figure 2).

Next, in order to achieve further liver-specific transgene expression, a liver-specific synthetic AHA promoter was incorporated in the Ad vector, creating Ad-AHAL2. Ad-AHAL2 achieved more than 100-fold higher luciferase expression in the neonatal liver compared to the other organs, although Ad-AHAL2 mediated about 5-fold lower transgene expression in the neonatal liver than Ad-L2 (Figure 2(b)).

In order to examine the Ad vector accumulation profile in the organs, the Ad vector genome copy numbers in the organs were measured by real-time PCR analysis. Lower levels of Ad vector genome were detected in all the neonatal organs, compared with the corresponding adult organs (Figure 2(c)). The highest levels of Ad vector genome were found in the liver among the neonatal organs examined. In spite of the approximately 10-fold higher luciferase expression in the neonatal heart than relative to the adult heart, approximately 2-fold lower levels of Ad vector accumulation were found in the neonatal heart than the adult heart. These results indicate that although the hepatotropism of Ad vectors is slightly weaker in neonatal mice than in adult mice, the liver is the primary organ transduced following systemic administration of an Ad vector even in neonatal mice. In addition, these results underscore that use of a liver-specific promoter augments the liver-specific transduction in neonatal mice.

### 3.2. Duration of Transgene Expression following Ad Vector Administration in Neonatal Mice

In order to assess the duration of Ad vector-mediated transgene expression in neonatal mice,* in vivo* bioluminescent imaging was carried out following Ad-AHAL2 administration. Although the Ad vector-mediated luciferase expression level was maintained from day 2 to day 7 after administration in neonatal mice, the intensity of luciferase bioluminescence was clearly decreased after day 14 and became almost equivalent to the background level on day 28 after administration (Figure 2(d)). This indicates that the Ad vector-mediated transgene expression in the liver gradually declines in neonatal mice.

### 3.3. Ad Vector-Mediated Toxicity in Neonatal Mice following Systemic Administration

**Figure 3:**
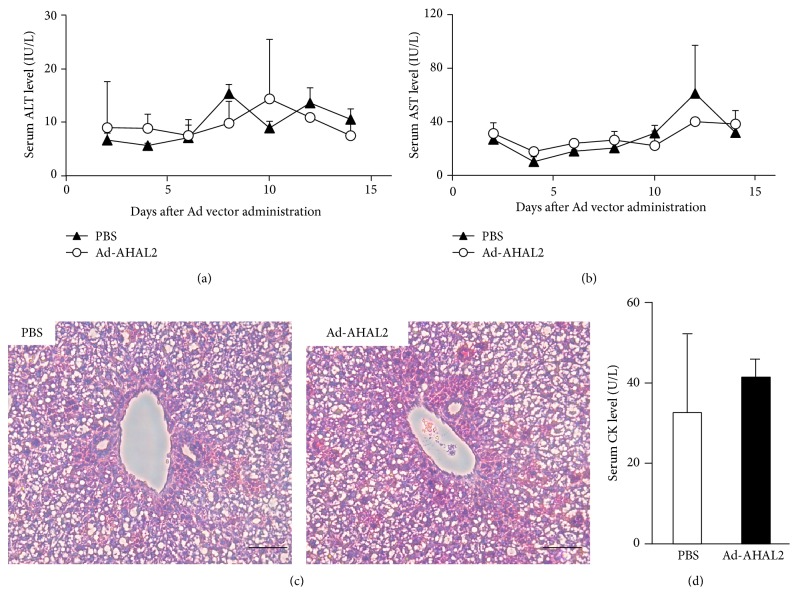
Ad vector-mediated toxicity in neonatal mice. (a, b) Serum ALT and AST levels in the neonatal mice following systemic administration of an Ad vector. Neonatal mice were administered Ad-AHAL2 at a dose of 5.9 × 10^11^ IFU/kg* via* retro-orbital sinus. Serum samples were collected at the indicated time points. (c) Liver sections of neonatal mice following systemic administration of PBS (left) and an Ad vector (right). The livers were isolated 48 h after Ad vector administration. Scale bars represent 100 *μ*m. (d) Serum CK levels in the neonatal mice following systemic administration of an Ad vector. Serum CK activity was measured 48 h after Ad-AHAL2 administration (*n* = 5 animals per group for a, b, and d and 2 for c).

**Figure 4:**
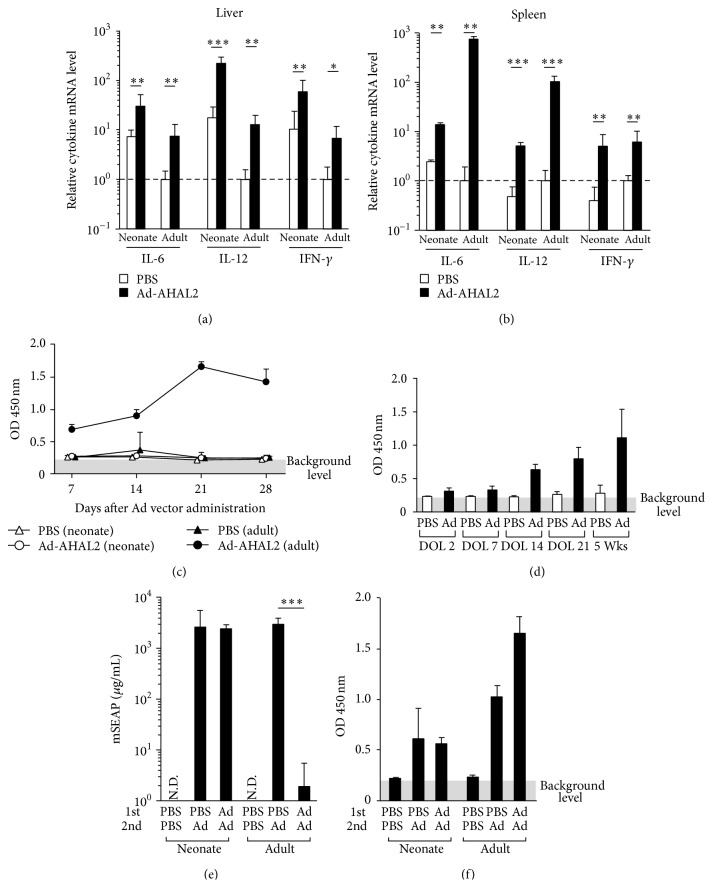
Immune responses in neonatal mice following systemic administration of an Ad vector. (a, b) Cytokine mRNA levels in the liver (a) and spleen (b) in neonatal and adult mice following systemic administration of an Ad vector. Mice were administered Ad-AHAL2 at a dose of 5.9 × 10^11^ IFU/kg* via* retro-orbital sinus. Organs were collected 3 h after Ad vector administration to recover total RNA. All values were normalized to 1 by the data of PBS-administered adult mice. (c) Anti-Ad antibody titers in the neonatal and adult mice following Ad vector administration. Mice were administered Ad-AHAL2 as described in (a). Serum samples were collected at each time point. Anti-Ad antibody levels were determined by ELISA. The background level represents the data of the PBST containing 5% ImmunoBlock. (d) Anti-Ad antibody production following systemic administration of an Ad vector in mice of different ages. Mice of different ages were administered Ad-AHAL2 as described in (a). Serum samples were collected 21 days after injection. Anti-Ad antibody levels were determined by ELISA. (e) mSEAP production after the second injection of Ad-AHAmSEAP. Mice received Ad-AHAL2 at a dose of 5.9 × 10^11^ IFU/kg* via* retro-orbital sinus. Ad-AHAmSEAP was administered 21 days after the first administration* via* tail vein at a dose of 5.9 × 10^11^ IFU/kg. Serum mSEAP levels were measured 48 h after injection of Ad-AHAmSEAP. (f) Anti-Ad antibody production in neonatal mice following the second injection of an Ad vector. Serum samples were collected 21 days after the second injection. Anti-Ad antibody levels were determined by ELISA (DOL, day of life; Wks, weeks) (*n* = 5 animals per group for a, b, e, and f: *n* = 3–5 animals per group for c: *n* = 4 animals per group for d).

In order to evaluate Ad vector-mediated toxicity in neonatal mice following systemic administration, the levels of two liver toxicity markers, serum alanine aminotransferase (ALT), and aspartate aminotransferase (AST) were examined following Ad vector administration. There were no significant differences in either the serum ALT or AST levels in neonatal mice between the Ad vector-treated group and PBS-injected group up to 14 days after administration at a dose of 5.9 × 10^11^ IFU/kg (Figures 3(a) and 3(b)), while we previously demonstrated that serum ALT and AST levels were elevated in adult mice following systemic administration of an Ad vector at this dose [13]. We further examined Ad vector-mediated liver toxicity by the histopathological examination of liver sections on day 2 following Ad vector administration. No apparent hepatotoxicity was observed in either the Ad vector- or PBS-injected group (Figure 3(c)). These data suggest that apparent hepatotoxicity is not induced in neonatal mice by Ad vector at a dose of 5.9 × 10^11^ IFU/kg. We also examined Ad vector-mediated cardiac toxicity in neonatal mice, because systemic administration of an Ad vector in neonatal mice resulted in efficient transduction in not only the liver but also the heart, as described above (Figures 2(a) and 2(c)). The result showed that the serum levels of CK, a cardiac toxicity marker, were not significantly elevated in the Ad vector-injected group compared with the PBS-injected group, suggesting that systemic administration of an Ad vector to neonatal mice did not induce apparent hepatic or cardiac toxicity (Figure 4(d)).

### 3.4. Ad Vector-Induced Immune Responses in Neonatal Mice

In order to evaluate Ad vector-induced innate immune responses in neonatal mice, real-time RT-PCR analysis was carried out to measure the cytokine mRNA levels in the liver and spleen at 3 h after injection in neonatal mice. The cytokine mRNA levels in the liver of PBS-injected neonatal mice were approximately 10-fold higher than those in the liver of PBS-injected adult mice (Figure 4(a)). Although Ad-AHAL2 induced 4- to 13-fold increases in the cytokine (IL-6, IL-12*β*, and IFN-*γ*) mRNA levels in not only the adult liver but also the neonatal liver, compared with treatment with PBS, the relative copy numbers of the cytokine mRNA in the neonatal liver were more than 4-fold higher than those in the adult liver following Ad vector administration. In the spleen, Ad-AHAL2 induced 5- to 13-fold elevations in the cytokine mRNA levels in neonatal mice, compared with PBS-injected mice; in the adult spleen, conversely, the cytokine mRNA levels were elevated 6- to 700-fold following injection of Ad-AHAL2 (Figure 4(b)). In addition, the adult spleen exhibited more than 20-fold higher relative copy numbers of IL-6 and IL-12*β* mRNA, compared with the neonatal spleen following Ad-AHAL2 injection. The relative copy numbers of IFN-*γ* mRNA in the neonatal and adult spleen were comparable following Ad-AHAL2 injection. These results indicate that systemic administration of an Ad vector significantly induces innate immune responses in neonatal mice, although the Ad vector-induced cytokine expression profiles in the liver and spleen are different between neonatal and adult mice.

Next, in order to examine anti-Ad antibody production in neonatal mice following Ad vector administration, the anti-Ad antibody titers were evaluated by ELISA. Following Ad vector injection, detectable levels of anti-Ad antibody titers were found in adult mice on day 7 post-injection, and the titers peaked on day 21 (Figure 4(c)). On the other hand, neonatal mice did not demonstrate a significant level of anti-Ad antibody titers up to day 28. We also examined anti-Ad antibody titers in serum following Ad vector administration in mice of different ages. As shown in Figure 4(d), anti-Ad antibody was detected in the serum of mice 14-days-old or older.

Next, in order to examine whether efficient transduction* via* repeated administration of an Ad vector was achieved in neonatal mice pretreated with an Ad vector, neonatal DOL 2 mice were injected first with Ad-AHAL2 and then, 21 days later, with Ad-AHAmSEAP. While the serum mSEAP levels were much lower in the preimmunized adult mice compared with the PBS preinjected group, almost equivalent levels of mSEAP expression were found between the neonatal mice pretreated with Ad-AHAL2 and those pretreated with PBS (Figure 4(e)). These results suggest that apparent induction of anti-Ad antibody does not occur in neonatal mice before DOL 7, and thus repetitive transduction could be performed* via* a second injection of Ad vector.

In order to examine anti-Ad antibody production following the second injection of an Ad vector, anti-Ad antibody titers were measured 21 days after the second injection. The second injection of Ad-AHAmSEAP induced comparable levels of anti-Ad antibody titers in both the neonatal mice pretreated with Ad-AHAL2 and those pretreated with PBS. In contrast, higher levels of anti-Ad antibody titers were produced in the adult mice pretreated with Ad-AHAL2, compared with the PBS-pretreated adult mice (Figure 4(f)). These results suggest that Ad vector administration at the neonatal stage does not induce immune tolerance against the Ad vector, although neonatal mice do not produce detectable levels of anti-Ad antibody following the first injection of an Ad vector.

## 4. Discussion

Although Ad vectors have been used in numerous preclinical studies employing mouse models of congenital disease [20–23], the properties of Ad vector transduction in neonates have not been fully examined. In this study, the properties of Ad vector-mediated transduction in neonatal mice following systemic administration were examined. The results showed that, not only in adult but also in neonatal mice, the levels of Ad vector accumulation and vector transgene expression were higher in the liver than in the other organs examined. The serotype 5 Ad vector used in this study is well known to exhibit hepatotropism [19]. The hepatotropism of an Ad vector is mainly mediated by FX. Although a previous study demonstrated that circulating FX levels in the blood in neonates were more than 2-fold lower compared with those in adults [24, 25], we found that pretreatment of an Ad vector with FX before injection did not significantly improve the hepatic transduction, suggesting that the lower levels of transgene expression in the liver were not mainly due to the low levels of FX in the blood. The low hepatic transduction in neonatal mice might have been due to the Ad vector being more widely distributed in peripheral tissues in neonatal mice than adult mice. The blood vessels in neonatal mice would be immature and leaky, which would allow an Ad vector to be widely distributed to in the peripheral tissues. We administered Ad vector* via* retro-orbital sinus in both neonatal and adult mice although systemic administration of an Ad vector in adult mice is usually performed* via* tail vein injection. We confirmed that almost equivalent or slightly higher levels of transgene expression in the organs were found in adult mice when an Ad vector was administered* via* retro-orbital sinus, compared with administration* via* the tail vein, indicating that the transduction profile of an Ad vector in mice* via* retro-orbital injection is almost identical to that* via* tail vein injection.

In contrast to the results for the liver, the neonatal heart exhibited about 10-fold higher transgene expression than the adult heart. It remains unclear why higher transduction efficiencies were obtained in the neonatal heart than in the adult heart. A previous study demonstrated that the expression levels of CAR, a primary Ad receptor, in the neonatal heart were comparable to those in the adult heart [26], suggesting that the CAR expression levels in the neonatal heart were not responsible for the efficient transduction in the neonatal heart. The expression levels of other infectious receptors for Ad, including *α*v-integrins and heparan sulfate, might have differed between the neonatal and adult heart.

Ad-L2, which contains a CMV promoter-driven luciferase expression cassette, mediated efficient luciferase expression not only in the neonatal liver but also in the neonatal heart. In contrast, liver-specific luciferase expression was achieved by use of a synthetic liver-specific promoter (AHA promoter). Liver-specific transgene expression is crucial to avoid side effects when the liver is targeted because the transgene expression in nontargeted organs may cause unexpected side effects. The AHA promoter-mediated transgene expression in the neonatal liver was about 5-fold lower than the CMV promoter-mediated transgene expression. We previously demonstrated that an AHA promoter showed significantly lower activity in the adult liver than a CMV promoter [27]. Intravenous administration of an Ad vector containing an AHA promoter resulted in relatively long-term transgene expression in the adult mice [13]; however,* in vivo* imaging analysis demonstrated that AHA promoter-mediated luciferase expression in the neonatal liver gradually declined and was almost equivalent to the background level on day 28. This is partly because the Ad vector copy numbers per cell are gradually diluted day by day due to the active hepatocyte proliferation in the neonatal liver. In our experiments, the body weight of the mouse increased by about 7-fold from day 2 to day 28 (data not shown).

Hepatotoxicity is one of the major side effects caused by systemic administration of an Ad vector. Previous studies reported that two peaks of ALT and AST elevation were observed at an early (days 2-3) and a later (days 8–12) time range in adult mice following systemic administration [13, 22, 28]. On the other hand, apparent elevation of serum ALT or AST levels was not found in neonatal mice at either the early or late time points. The latter peak of the hepatotoxicity was mainly caused by cellular immunity against Ad proteins [29, 30]. Ad vector-mediated hepatotoxicity was not induced on days 8–12 because the level of acquired immunity was still very low in neonatal mice. On the other hand, Ad vector-induced hepatotoxicity at early time points is caused by innate immunity [31]. Nonetheless, although Ad vector-mediated innate immunity was induced even in the neonatal liver and spleen 3 h after injection, the mRNA levels of IL-6 and IL-12 in the neonatal spleen were more than 10-fold lower than those in the adult spleen following Ad vector administration, which would count for the lack of hepatotoxicity at the early time points. The spleen plays a major role in Ad vector-induced innate immune response [32]. The mRNA levels of the cytokines in the liver of PBS-injected neonatal mice were significantly higher than those in the liver of PBS-injected adult mice, probably because there were larger numbers of blood cells in the neonatal liver than the adult liver due to extramedullary hematopoiesis in the neonatal liver.

The anti-Ad antibody titers were much lower in neonatal mice than adult mice following Ad vector administration, which allowed efficient transgene expression following the second injection of an Ad vector. These properties are highly attractive for gene therapy in neonates because immune responses against viral vectors and transgene products hamper efficient transduction in adults. However, significant levels of anti-Ad antibodies were produced following the second injection of an Ad vector in the neonatal mice, indicating that immune tolerance was not induced. This is disadvantageous for gene therapy; however, patients are protected from natural infection with Ads.

In summary, we demonstrated that efficient transduction in the neonatal liver can be achieved by systemic administration of an Ad vector without apparent hepatotoxicity. The use of a liver-specific promoter further augmented the liver-specific transgene expression in neonatal mice. These findings should provide valuable information for the use of Ad vector-mediated transduction in both gene therapies for neonates and basic researches using neonatal animals.

## Supplementary Material

Supplementary figure 1: In order to examine whether low levels of coagulation factor X (FX) in the neonatal blood affect the low hepatic transduction of an Ad vector in neonatal mice, we mixed Ad-L2 with human FX before administration and examined transduction efficiencies of an FX-pretreated Ad vector in the neonatal liver. We found that pretreatment of Ad-L2 with FX before injection did not improve the transduction efficiencies of Ad-L2 in the liver of neonatal mice.
Supplementary figure 2: In order to examine whether injection routes of an Ad vector affect transduction efficiency in the organs, Ad-L2 was administered to adult mice via tail vein injection and retro-orbital injection. We did not find significant differences in the transduction profiles of Ad-L2 in adult mice following tail vein injection and retro-orbital injection.

## Figures and Tables

**Table 1:** Ad vectors used in this study.

Vector name	Promoter	Transgene	vp/IFU
Ad-L2	CMV	Firefly luciferase	7.6
Ad-AHAL2	AHA	Firefly luciferase	11.7
Ad-AHAmSEAP	AHA	mSEAP	13.4

CMV, cytomegalovirus promoter; AHA, a synthetic promoter composed of an apolipoprotein E enhancer, the hepatocyte control region, and a human *α*1-antitrypsin promoter; mSEAP, murine secreted embryonic alkaline phosphatase.
