# Supplementary material for: Evaluation of Transduction Properties of an Adenovirus Vector in Neonatal Mice
Source: Biomed Res Int. 2015 May 13;2015:685374. doi: 10.1155/2015/685374 (PMC4444570; doi:10.1155/2015/685374)
Supplement: Supplementary file 1 — Supplementary figure 1: In order to examine whether low levels of coagulation factor X (FX) in the neonatal blood affect the low hepatic transduction of an Ad vector in neonatal mice, we mixed Ad-L2 with human FX before administration and examined transduction efficiencies of an FX-pretreated Ad vector in the neonatal liver. We found that pretreatment of Ad-L2 with FX before injection did not improve the transduction efficiencies of Ad-L2 in the liver of neonatal mice. Supplementary figure 2: In order to examine whether injection routes of an Ad vector affect transduction efficiency in the organs, Ad-L2 was administered to adult mice via tail vein injection and retro-orbital injection. We did not find significant differences in the transduction profiles of Ad-L2 in adult mice following tail vein injection and retro-orbital injection. [file 685374.f1.pdf]

Supplementary figures and figure legends

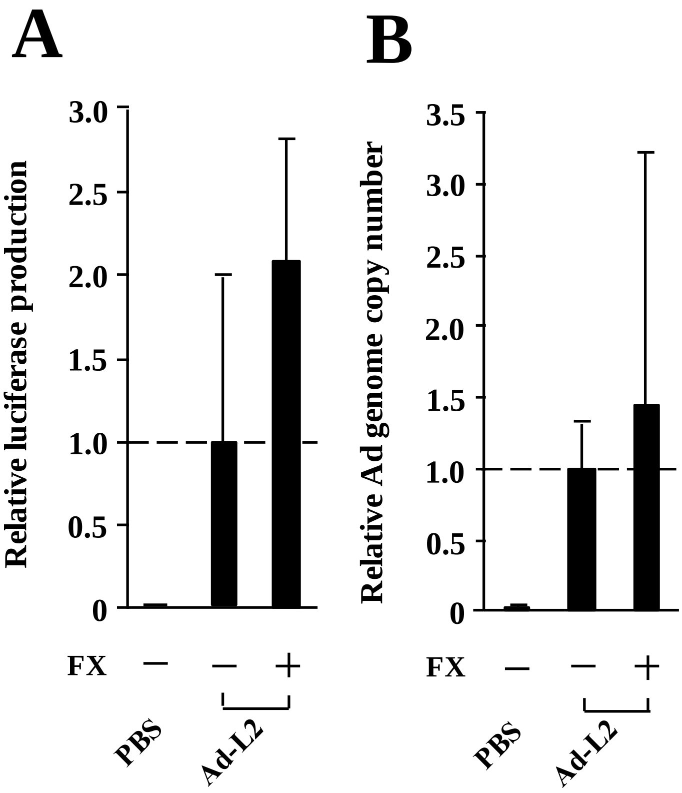

Fig. S1 Transduction efficiencies of an FX-pretreated Ad vector in the neonatal liver. Ad-L2 was mixed with human FX before administration. The mixture of Ad-L2 and human FX was administered *via* retro-orbital sinus at doses of $5.9x10^{11}$ IFU/kg for Ad-L2 and 1.1 mg/kg for human FX. Luciferase production (A) and Ad genome copy numbers (B) were examined in the neonatal liver 48 h after Ad vector administration. All values were normalized to 1 by the data of Ad-L2-administered neonatal mice.

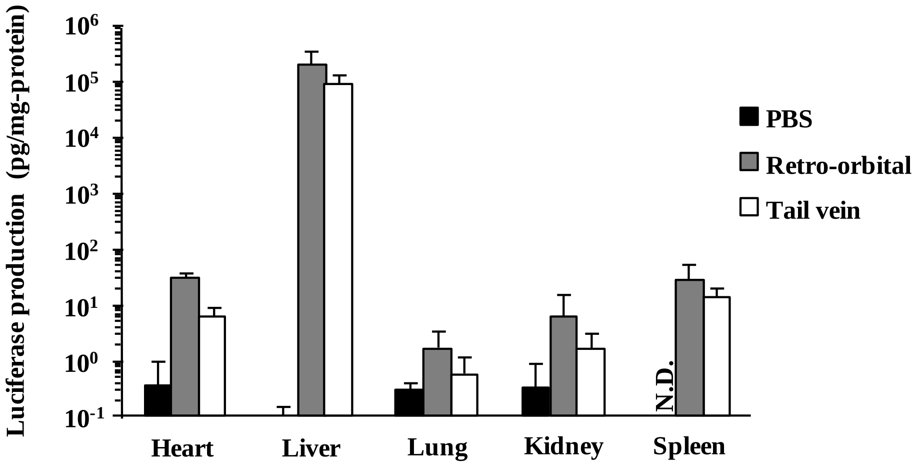

Fig. S2 Ad vector-mediated transgene expression in the organs *via* retro-orbital sinus and tail vein administration.

Adult mice were administered Ad-L2 at a dose of 5.9×10^{11} IFU/kg *via* retro-orbital sinus and the tail vein. Luciferase production was examined in each organ 48 h after Ad vector administration.
